# Insights from Computational Modeling in Inflammation and Acute Rejection in Limb Transplantation

**DOI:** 10.1371/journal.pone.0099926

**Published:** 2014-06-13

**Authors:** Dolores Wolfram, Ravi Starzl, Hubert Hackl, Derek Barclay, Theresa Hautz, Bettina Zelger, Gerald Brandacher, W. P. Andrew Lee, Nadine Eberhart, Yoram Vodovotz, Johann Pratschke, Gerhard Pierer, Stefan Schneeberger

**Affiliations:** 1 Department of Plastic, Reconstructive and Aesthetic Surgery, Innsbruck Medical University, Innsbruck, Austria; 2 Language Technologies Institute, Carnegie Mellon University, Pittsburgh, Pennsylvania, United States of America; 3 Division of Bioinformatics, Biocenter, Innsbruck Medical University, Innsbruck, Austria; 4 Department of Immunology, University of Pittsburgh, Pittsburgh, Pennsylvania, United States of America; 5 Department of Visceral, Transplant and Thoracic Surgery, Innsbruck Medical University, Innsbruck, Austria; 6 Department of Pathology, Innsbruck Medical University, Innsbruck, Austria; 7 Department of Plastic and Reconstructive Surgery, Johns Hopkins University School of Medicine, Baltimore, Maryland, United States of America; UNIFESP Federal University of São Paulo, Brazil

## Abstract

Acute skin rejection in vascularized composite allotransplantation (VCA) is the major obstacle for wider adoption in clinical practice. This study utilized computational modeling to identify biomarkers for diagnosis and targets for treatment of skin rejection. Protein levels of 14 inflammatory mediators in skin and muscle biopsies from syngeneic grafts [n = 10], allogeneic transplants without immunosuppression [n = 10] and allografts treated with tacrolimus [n = 10] were assessed by multiplexed analysis technology. Hierarchical Clustering Analysis, Principal Component Analysis, Random Forest Classification and Multinomial Logistic Regression models were used to segregate experimental groups. Based on Random Forest Classification, Multinomial Logistic Regression and Hierarchical Clustering Analysis models, IL-4, TNF-α and IL-12p70 were the best predictors of skin rejection and identified rejection well in advance of histopathological alterations. TNF-α and IL-12p70 were the best predictors of muscle rejection and also preceded histopathological alterations. Principal Component Analysis identified IL-1α, IL-18, IL-1β, and IL-4 as principal drivers of transplant rejection. Thus, inflammatory patterns associated with rejection are specific for the individual tissue and may be superior for early detection and targeted treatment of rejection.

## Introduction

Rejection in Vascular Composite Allotransplantation (VCA) is characterized by an inflammatory cell-mediated cytotoxic process, which progressively harms the epidermis and the junction between dermis and epidermis, unless reversed or prevented by immunosuppression. Understanding the immune signaling patterns of skin rejection would enable the development of targeted and local therapy with fewer side effects.

The current gold standard for the diagnosis of rejection is histological evaluation of tissue biopsies according to the BANFF 2007 working classification [1]. Assessing rejection by histology suffers from latency between initiation of tissue damage and diagnosis. Often, histological signs of skin rejection have been found in protocol biopsies despite absence of clinical signs of rejection. As stated previously by our group and others, the histopathological alterations associated with rejection are not specific, but rather similar to several common inflammatory dermatoses [2], [3] or the result of an inflammatory trigger [4]. The differential diagnosis between skin rejection, infection and unspecific inflammation can be challenging in hand- and especially face transplantation. The conditions are similar in their appearance and may interfere with or trigger each other [5].

Traditional methods are of limited value for elucidation of how the immune/inflammatory response in the skin affects VCA through intricate signaling patterns and context-dependent behaviors. Advanced computational methods such as language technologies and machine learning offer significant advances in deciphering complex processes from other areas of science [6], [7].

We hypothesized that mechanisms of rejection in VCA are tissue specific and can be detected in advance of gross histological damage by assessing leukocyte expression patterns with advanced computational tools. Based on our findings, promising diagnostic markers for skin and muscle rejection as well as possible targets for new therapeutic interventions in VCA have been identified.

## Methods

### Experimental design

All animal procedures, care, and housing were reviewed and approved by the Institutional Animal Care and Use Committee (IACUC) of the University of Pittsburgh (protocol number: 0808858B-2), and followed the National Institutes of Health guidelines for the care and use of laboratory animals. A summary of the cohorts and the conditions they represent are presented in Table 1. Limb transplantations including skin, muscle, bone and vessels, were performed as per a standardized technique between eight- to ten-week-old male Brown-Norway (BN) and Lewis rats (LEW) weighing 200–250 g with (group 3) or without (group 1) immunosuppression and compared with untreated isografts (group 2) [8]. Animals were anesthetized with a combination of xylazine (Xylasol, 5 mg/kg) and ketamine (Ketavet, 100 mg/kg), injected intramuscularly.

**Table 1:** Study groups and design.

Group (Name)	Animals per group	Donor	Recipient	Treatment	Samples per time point (POD 3,5,7,9)	Samples per animal	Tissue
1 (ATC)	10	BN	LEW	none	5	3	Skin & Muscle
2 (ISO)	10	LEW	LEW	none	5	3	Skin & Muscle
3 (TAC)	10	BN	LEW	Tacrolimus*	5	3	Skin & Muscle

* 1 mg/kg/day (for 11 days).

### Assessment of rejection

Animals were inspected daily for signs of rejection. Skin rejection was classified per appearance: Grade 0 – no signs of rejection; Grade I – erythema of the transplanted leg, Grade II – erythema and edema, Grade III – epidermolysis of the transplanted skin, Grade IV – mummification of the leg (limb necrosis). In untreated animals (allografts, ATC), rejection occurs after 3-4 days (Grade I rejection) and progresses to Grade IV rejection between day 9 and 11. Samples from allograft skin and muscle were collected at postoperative days (POD) 3, 5, 7, 9 and 11 in all three groups in a staggered fashion. To out rule an impact of the trauma an the readout of subsequent tissue samples, biopsies were taken from sites distant to another on days 3, 5, 7 and 11, or days 5, 9 and 11 (see Table 2). Since all animals showed mummification of the graft on POD 11 with super infection in some, samples from this time point were excluded from the study.

**Table 2:** Appearance (Grading for skin rejection: I–IV) of each allograft on POD 1–11.

Animals ID	POD (Postoperative Day)										
	1	2	3	4	5	6	7	8	9	10	11
ATC 1	0	0	0	I	**I**	II	II	III	**III**	IV	**IV**
ATC 2	0	0	0	I	**II**	II	III	III	**IV**	IV	**IV**
ATC 3	0	0	**0**	0	**I**	II	**III**	III	IV	IV	**IV**
ATC 4	0	0	**0**	0	**I**	II	**II**	III	IV	IV	**IV**
ATC 5*	0	0	**0**								
ATC 6	0	0	**0**	0	I	II	**II**	III	**III**	IV	**IV**
ATC 7	0	0	**0**	I	II	II	**III**	III	**IV**	IV	**IV**
ATC 8	0	0	0	I	**II**	II	**II**	III	IV	IV	**IV**
ATC 9	0	0	0	I	**II**	II	II	III	**IV**	IV	IV
ATC 10	0	0	**0**	I	I	II	**II**	III	III	IV	IV
ATC 11	0	0	0	I	**II**	II	II	III	**III**	IV	**IV**

*This animal died on POD 3 during the anesthesia. Bold numbers mark biopsy timepoints (skin and muscle) for each animal.

The size of each tissue biopsy per chosen time point was approximately 25×10 mm. This tissue sample was divided into 3 identical parts for further analyses. One biopsy part (piece) was fixed in 10% buffered formalin and processed routinely for hematoxilyn and eosin (H&E) staining. Sections were evaluated for lymphocytic infiltration, dermal/epidermal interphase reaction, dermal-epidermal separation and necrosis by a pathologist in a blinded fashion. The other biopsy parts were preserved in RNALater for protein analysis and RNA isolation.

### Protein isolation and protein expression analysis

Proteins from skin and muscle samples were isolated using a disperser (T10, basic ULTRA-TURRAX, IKA, Germany) with 1 ml 1 x Cell Lysis Buffer (Cell Signaling, Danvers, USA) per sample on ice. Proteins were quantified after homogenizing using the BCA Protein Assay Kit according to the manufacturer's protocol.

Inflammatory mediator expression at the protein levels was measured using the Luminex inflammatory mediator bead set (RCYTO-80K-PMX-14-plex Milliplex Map Kit from Millipore, Billerica, MA) that included interferon (IFN)-γ, IL-1α, IL-1β, IL-2, IL-4, IL-5, IL-6, IL-10, IL-12p70, IL-18, monocyte chemotactic protein (MCP-1), GRO/KC, TNF-α, and granulocyte-macrophage colony stimulating factor (GM-CSF) in a Luminex 100 IS (Luminex Corporation, USA) and analyzed by xPonent 3.1 Rev.2 Software (Luminex Corporation, USA). Results for each of the 14 analytes were read in pg/ml and subsequently normalized to total mass of sample protein (pg inflammatory mediator/mg protein) by multiplying with 0.025 ml standard sample volume and dividing with 0.1 mg added total protein for each sample. Any analytes indicating a concentration above 20,000 pg/µl were excluded from analysis (NA) as being outside the linear range of the Luminex assay.

### Statistical and computational analysis

Analyses were performed with the statistical framework R 2.13.1 using packages including *stats, nnet*, *multtest, MASS, beeswarm, randomForest.* The non-parametric Kruskal-Wallis test was used to compare mediator abundance among groups 1-3 (ATC, ISO, TAC) at POD 3/POD 5. The Wilcoxon rank-sum test was used to identify the tissue levels of those mediators whose levels varied significantly between animals exhibiting rejection (ATC) and animals treated with tacrolimus to prevent rejection (TAC) in the early postoperative phase (POD 3/POD 5), as well for selected inflammatory mediators at POD 3. All p-values were adjusted for multiple hypothesis testing based on the false discovery rate (FDR) [9].

The multivariate extension of the one-way analysis of variance (MANOVA) or the discriminant function analysis (based on the same formulation as MANOVA with inflammatory mediators as independent variables and the rejection group as dependent variable in the latter case) are used to determine the coefficients of the two orthogonal discriminant vectors (DV), which enable maximal separation of the groups. As a measure of contribution to these vectors for each mediator, the sum of the absolute values of the respective coefficients (loadings) were used and related to the overall sum. Pillai's trace statistic was used to test for the differences in the vectors of means. The mediators showing significant differences among the 3 groups and with >2.5% contribution to the DVs in both skin and muscle were subjected to multinomial (logistic) regression analyses. A model for skin and one model for muscle were selected based on minimal Akaike information criterion [AIC]. Classifier performance was assessed using a 8-fold cross validation procedure and visualized using confusion tables. Accuracy was defined as 1- misclassification rate and Welch's test was used to test the differences between the mean of the number of true predicted and the mean of the sum of the respective number of false predicted.

To assess similarity of inflammatory mediator levels among different time points and tissue types, complete-linkage hierarchical clustering was performed and visualized as heat map using Genesis [10] based on mean-centered log2-transformed profiles. For this analysis, the mean value for each group and mediator was used.

A Random Forest (RF) [11] approach was used for classification of the rejection group (ATC, ISO, TAC) including all time points. This method was also used to identify those mediators most important for classification or diagnosis. Classifier performances were assessed by confusion tables and the out-of-bag (OOB) error rate. Principal Component Analysis (PCA) was used to rank most variable (important) mediators and potential therapeutic target candidates [12]. PCA reduces a multidimensional dataset to a few principal components, which account for the most variability in the dataset. The underlying hypothesis is that a mediator which changes during a process is important to that process [12], [13]. In this analysis, the data were combined from skin and muscle, mean centered, and variance scaled. Components sufficient to capture at least 70% of total data variance observed were included.

## Results

### Progression of rejection

#### Appearance

On postoperative day (POD) 3, none of the allografts (n = 10) showed signs of rejection, on POD 5, 50% of the allografts displayed Grade I rejection and 5 animals (50%) II rejection. On postoperative day 7, rejection Grade II was present in 7 animals (70%) and rejection Grade III in 3 animals (30%). On day 9, 4 animals displayed Grade III rejection and 6 animals Grade IV. None of the syngeneic controls or tacrolimus treated animals showed any signs of skin rejection/inflammation (Table 2).

#### Histological evaluation of skin biopsies

**Figure 1:**
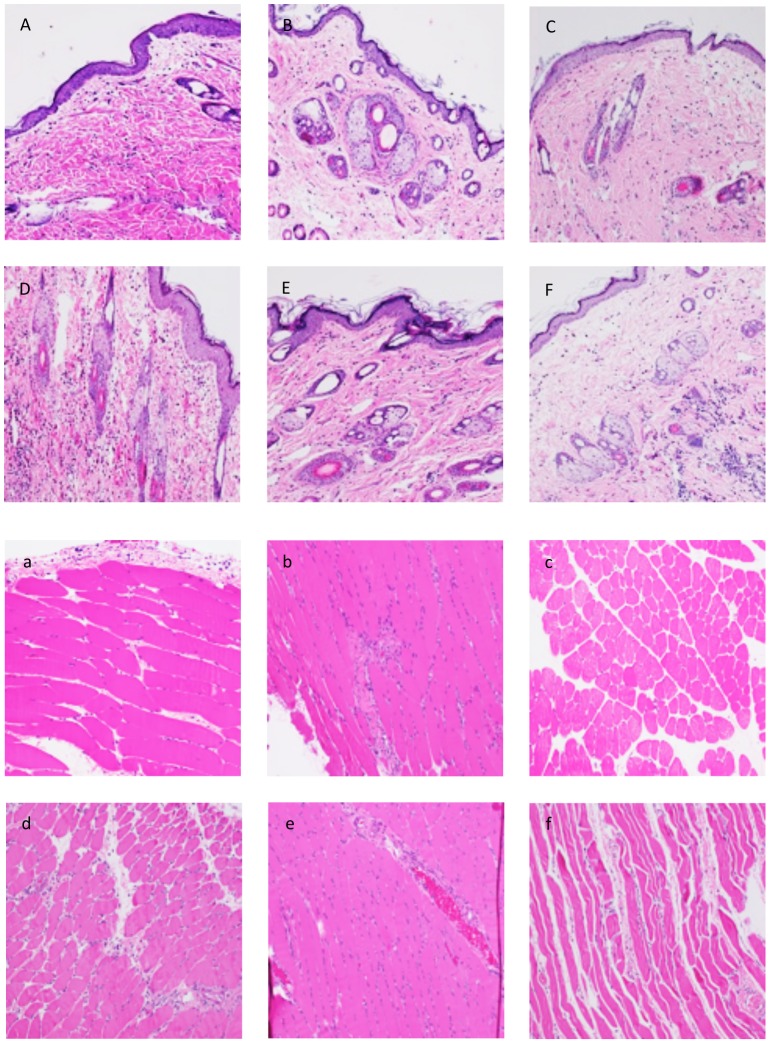
Histological evaluation of skin and muscle rejection in the early postoperative phase (POD 3 and 5). (A–C) Skin sample taken on POD 3 from an allograft without immunosuppression (A), from an isograft (B) and an allograft under TAC (C) showing no/rare inflammatory response (Grade 0 rejection). (D–F) Skin biopsies taken on POD 5 from a rejecting animal (D) displaying Grade 1 rejection, from an isograft (E) showing no/rare inflammatory response and a TAC treated allograft (F) characterized by a mild inflammatory response (Grade 0-I) in the deep dermis. (a–c) Muscle sample taken on POD 3 from an allograft without immunosuppression (a), from an isograft (b) and an allograft under TAC (c) showing no/rare inflammatory response (Grade 0 rejection). (d–f) Skin biopsies taken on POD 5 from a rejecting animal (d) displaying a mild inflammatory response (Grad 0-I rejection), from an isograft (E) and a TAC treated allograft (f) showing no/rare inflammatory response.

Histological evaluation of allograft skin biopsies on day 3 showed no or minimal inflammatory infiltrates (Grade 0) in six animals and a mild perivascular infiltrate (Grade I) in one biopsy. No tacrolimus-treated animals showed any signs of rejection while a mild perivascular infiltration was seen in one of the syngeneic control animal. On POD 5, biopsies from allogeneic transplants displayed Grade I rejection in three animals, as well as a moderate to severe perivascular inflammation with or without mild epidermal and/or adnexal epidermal dyskeratosis or apoptosis (Grade II) in three animals. One biopsy taken from the allogeneic group showed a severe skin rejection (Grade III), with dense inflammation and epidermal involvement with epithelial apoptosis, dyskeratosis and keratinolysis on POD 5. No inflammatory response was observed in isografts on POD 5, but two out of five biopsies in the tacrolimus treated animals did show an inflammatory infiltrate. On POD 7, allogeneic animals showed Grade III rejection and, one skin biopsy taken from the isograft group and one from the tacrolimus group displayed a moderate inflammation corresponding with Grade I/II rejection. At the endpoint, all samples from allografts showed rejection Grade III and one biopsy (n = 5) taken from the isografts displayed the characteristics of Grade I rejection. Two out of five biopsies in the tacrolimus group showed a mild rejection (Grade I) and one biopsy was classified as Grade II rejection (Table 3 and Figure 1).

**Table 3:** Histological evaluation of skin biopsies from postoperative (POD) 3 to 9.

SKIN	POD 3				POD 5				POD 7				POD 9			
GRADE	0	I	II	III	0	I	II	III	0	I	II	III	0	I	II	III
ATC	6	1				3	3	1				6				5
ISO	3	1			4				4		1		4	1		
TAC	5				3	2			4	1			2	2	1	

Number of biopsies taken on each postoperative day (POD 3,5,7,9) in skin according to their histological grading (Grad 0-III) based on H&E staining.

#### Histological evaluation of muscle biopsies

H&E stains from allograft muscle samples showed no or rare inflammatory cells on POD 3 in four biopsies and Grade I rejection in two biopsies. Muscle biopsies from syngeneic controls showed a mild infiltration in two biopsies, severe inflammation similar to rejection Grade II in one graft, and no inflammatory response at this time point in two animals. On POD 5, two biopsies from the allogeneic transplants were classified as Grade 0 rejection, three samples as Grade I and one muscle biopsy as Grade II rejection, four biopsies from the isografts showed no or rare inflammation and only one sample a mild inflammation.

On POD 7, all biopsies (n = 5) from allografts displayed Grade I rejection and only one biopsy from an isografts showed a mild muscle inflammation on both day 7 and 9. Allograft muscle biopsies on day 9 showed rejection Grade I (n = 1, Grade II (n = 2) or Grade III (n = 2). Tacrolimus treated animals did not show any signs of rejection except for one animal displaying mild inflammation in the muscle on POD 9 (Table 4 and Figure 1).

**Table 4:** Histological evaluation of muscle biopsies from postoperative (POD) 3 to 9.

MUSCLE	POD 3				POD 5				POD 7				POD 9			
GRADE	0	I	II	III	0	I	II	III	0	I	II	III	0	I	II	III
ATC	4	2			2	3	1			5				1	2	2
ISO	2	2	1		4	1			4	1			4	1		
TAC	5				5				5				4	1		

Number of biopsies taken on each postoperative day (POD 3,5,7,9) in muscle according to their histological grading (Grad 0-III) based on H&E grading. The first biopsy was taken from the lateral proximal part of the thigh, the second one from the lateral distal thigh, the third one from the ventral thigh and the last one from the medial part of the thigh.

### Significant differences of inflammatory mediator levels at early postoperative time points among the different transplant models

**Figure 2:**
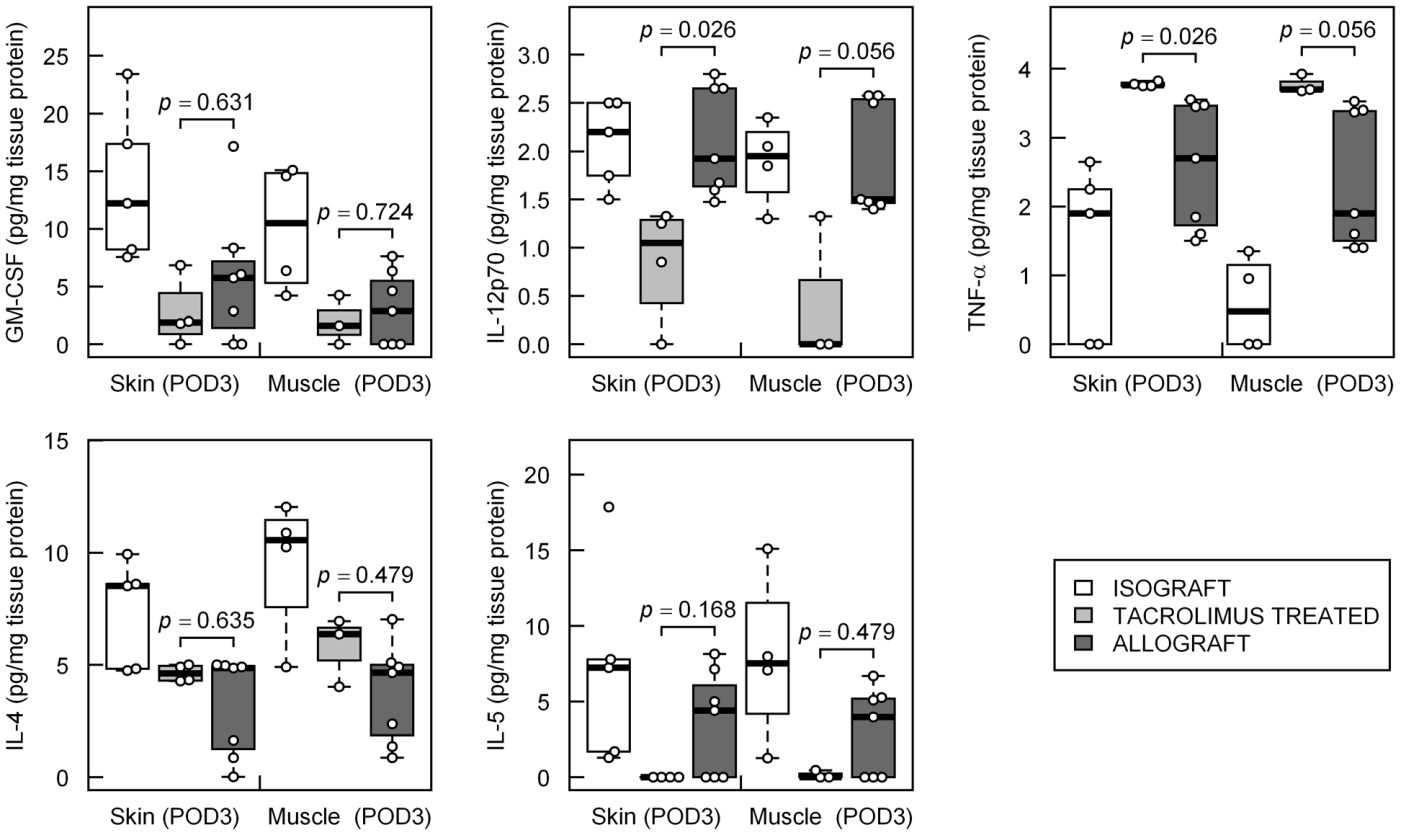
Distribution of inflammatory mediator levels at the earliest postoperative measurements (POD 3) in rat limb transplantation models. Adjusted p-values from Wilcoxon rank-sum test between the rejection group (ATC) vs. Tacrolimus treated group (TAC) for selected inflammatory mediators, which were tested to be included in a prediction model by multinomial logistic regression analysis, are presented.

We examined the levels of 14 inflammatory analytes in skin and muscle biopsies of allogeneic, syngeneic and immunosuppressed hind limb transplants at different postoperative days (POD 3, 5, 7, and 9) with a focus on the early postoperative phase where no histological alterations were observed (POD 3 and 5, Figure 2 and Figure S2). Non-parametric univariate analysis of the inflammatory mediators from skin and muscle were performed. Five inflammatory mediators (GM-CSF, IL1-α, IL-4, IL-12p70, IL-5, TNF-α) were significantly different at least in one group (adjusted p<0.05; Kruskal-Wallis test [KW]) in both skin and muscle. IL-12p70 and TNF-α were highly significantly different in the allograft versus the tacrolimus-treated animals (adjusted p<0.005; Wilcoxon-rank sum test [WR]; Table 5).

**Table 5:** Statistical analysis for all 14 inflammatory mediator levels in skin and in muscle at early postoperative time points (combined group of POD 3 and POD 5).

	Skin POD 3/5			Muscle POD 3/5		
	*p_KW_*	*p_WR_*	Partition on DVs [%]	*p_KW_*	*p_WR_*	Partition on DVs [%]
**GM-CSF**	**0.0018**	0.3260	**4.26**	**0.0083**	0.6178	**2.60**
IL-1α	**0.0244**	1.0000	0.01	**0.0105**	0.0773	0.01
MCP-1	**0.0011**	**0.0026**	0.72	**0.0466**	0.3673	0.79
**IL-4**	**0.0040**	0.3421	**24.21**	**0.0194**	0.5312	**18.68**
IL-1β	0.2042	0.1577	0.10	0.8251	0.7577	0.09
IL-2	0.2042	0.2868	0.83	0.1779	0.5312	0.29
IL-6	0.1322	0.2824	0.27	0.5127	0.6178	0.29
IL-10	0.2777	0.2805	0.55	0.8251	0.8657	0.39
**IL-12p70**	**0.0019**	**0.0013**	**15.65**	**0.0030**	**0.0025**	**31.59**
**IL-5**	**0.0022**	**0.0123**	**4.85**	**0.0010**	0.2112	**5.03**
IFN-γ	0.2956	0.3729	**5.67**	0.2562	0.3619	1.90
IL-18	0.3462	0.2868	0.03	**0.0425**	0.7498	0.03
GRO-KC	**0.0040**	**0.0021**	0.27	0.5127	0.5312	0.11
**TNF-α**	**1.7×10^−4^**	**0.0013**	**42.59**	**0.0001**	**0.0025**	**38.21**

Most promising inflammatory mediators for classification of rejection, based on early identification before histological manifestation of rejection is evident, are selected (*p_KW_*<0.05 and *Partition on DVs* >1%, symbols in bold) and subjected to multinomial logistic regression analyses. For all measured inflammatory mediators adjusted p-values from Kruskal-Wallis test (KW) of overall equality between all 3 cohorts (ISO, TAC, ATC) and Wilcoxon rank-sum test (WR) for comparison between the rejection group (ATC) and the Tacrolimus treated group (TAC) as well as partition of each inflammatory mediator on the two discriminant vectors (DVs), which maximizes the separation between the 3 rejection groups, resulting from discriminant function analysis, are given.

In Figure 2, the levels of the concentrations of these inflammatory mediators at POD 3 of all three groups within skin and muscle are depicted. Significantly higher concentrations of IL-12p70 in the skin from the allograft animals compared to the tacrolimus-treated animals (adjusted p = 0.026) as well as lower abundance of TNF-α in the allogeneic compared to the immunosuppressed transplants (adjusted p = 0.026) were detected. In muscle, numerical differences did not reach statistical significance (adjusted p = 0.056 in both cases).

For identification of inflammatory mediators with the highest predicting value, at early time points, multivariate analyses were performed. We fitted multivariate analysis of variance (MANOVA) models which resulted in *p* = 8.9×10^−10^ for skin and *p* = 3.4×10^−5^ for muscle from Pillai's trace statistic. Using these models, a functional discriminant analysis was performed. Mediators with the greatest absolute coefficients in the two resulting discriminant vectors might contribute most to the separation of groups (Table 5). The partition to the discrimination vectors for GM-CSF, IL-4, IL-12p70, IL-5, and TNF-α were >2.5% in both tissues. We selected these inflammatory mediators as promising biomarkers candidates for further analyses.

### Inflammatory mediators at early time points discern the procedure to which animals were subjected

A major goal of this study was to determine markers for early and accurate diagnosis of skin rejection in advance of major histological alterations. We applied several multivariate multinomial logistic regression models in skin and muscle samples for the classification of the three study groups at early postoperative time points (POD 3 and POD 5) using a combination of the selected inflammatory mediators (GM-CSF, IL-4, IL-12p70, IL-5, and TNF-α) as independent variables and study group as outcome. Based on the Akaike information criterion (AIC), which is a measure of the trade-off between the complexity of the model, i.e. number of variables, and the goodness of fit, optimal models for differentiation of the study groups could be found in skin rejection (based on IL-4, IL-12p70, and TNF-α: AIC = 16.0; residual deviance = 1.7×10^−4^) and in muscle (based on IL-12p70 and TNF-α: AIC = 12.0; residual variance = 1.6×10^−4^). The coefficients of the inflammatory mediators in the logistic regression models and classifier performance as assessed by 8-fold cross validation are detailed in Table 5. The prediction accuracy within skin was 87.1% and in muscle 100%, as derived from classification tables. A pairwise multivariate logistic regression analysis between the study groups (ATC vs. TAC, TAC vs. ISO, ISO vs. ATC) and applying a leave-one-out cross validation strategy resulted in an area under curve (AUC) from receiver operating characteristics (ROC) for skin of 0.5, 0.69, and 0.86 and for muscle of 1.0, 1.0, and 1.0, indicating a substantially better discrimination than by chance (AUC = 0.5) (Table 6).

**Table 6:** Results for multinomial logistic regression models in skin and muscle for classification of the rejection groups based inflammatory mediator/chemokine levels at early postoperative time points (POD 3/5) in rat transplantation models.

	Skin POD3/5			Muscle POD3/5		
ln(p(*TAC*)/p(*ATC*))	-6.7-2.6*IL4-61.3*IL12p70+25.6*TNFa			-6.9-18.8*IL12p70+11.3*TNFa		
ln(p(*ISO*)/p(*ATC*))	-1094.8+112.6*IL4+250.4*IL12p70-28.6*TNFa			-10.0+25.1*IL12p70-27.0*TNFa		
	ATC^pred^	TAC^pred^	ISO^pred^	ATC^pred^	TAC^pred^	ISO^pred^
ATC	12	0	1	13	0	0
TAC	0	8	0	0	7	0
ISO	3	0	7	0	0	9
	Accuracy = 87.1% *p* = 0.020			Accuracy = 100% *p* = 0.031		

Best prediction models for skin and muscle and their respective logistic prediction functions and classifier performance including confusion table (assessed by an 8-fold cross validation procedure) are summarized.

### Inflammatory mediator profiles are similar within each group

**Figure 3:**
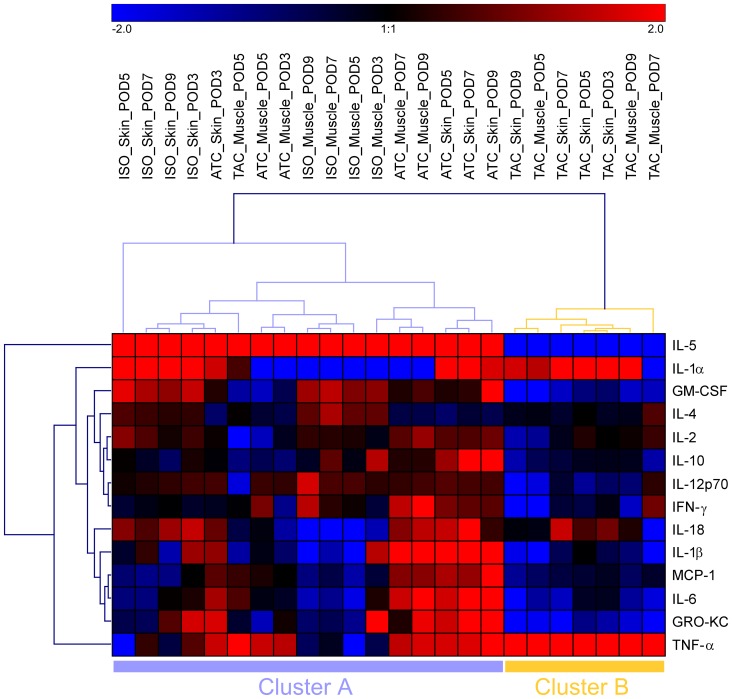
Similarity of sample groups and association between inflammatory mediators in rat limb transplantation models based on their profiles of mean levels in each condition. Heatmap as a result of complete linkage hierarchical clustering on log2-transformed and mean centered data. Log2-fold differences against the respective mean levels of each inflammatory mediator are color coded (red means higher inflammatory mediator levels and blue means lower inflammatory mediator levels than the mean levels of the respective inflammatory mediator according to the color scheme at the top). Dendrograms (trees) show similarity between different conditions and different inflammatory mediator profiles, respectively.

Hierarchical clustering was performed for all inflammatory mediators in the three experimental groups. Each row of the data matrix corresponds with one of the 14 inflammatory mediators, and each column corresponds with a group. The log_2_-transformed and mean-centered values (mean concentrations from each condition) are visualized as a heat map, with color codes shown in the color bar (Figure 3). The dendrogram on the x-axis shows the similarities among the samples. As expected, the rejection group (ATC skin and muscle samples), especially samples from later time points with pronounced histological changes, exhibit a completely different mediator clustering pattern vs. the control groups, with high abundance of IL-5, IL-18, IL-1β, MCP-1, IL-6, GRO-KC, and TNF-α. In TAC-treated animals, only IL-1α and TNF-α were highly abundant, whereas the expression of all other mediators assessed appeared suppressed.

The separation of the tacrolimus-treated group was also evident from the cluster analysis. Within sample cluster A (17 samples), 5.9% were from the tacrolimus and 94.1% were from study group ISO/ATC. In contrast, in cluster B (7 samples), 100% were from tacrolimus and 0% from the ISO/ATC groups. A Fisher's exact test (*p* = 2.3×10^−5^) suggested that the distribution between clusters A and B was not random. Profiles of MCP-1, IL-4, IL-1β, and IL-6 characterize rejecting grafts, whereas GM-CSF and IL-4 characterize isografts. This was also indicated by an absolute value of point-biserial correlation coefficient >0.6 comparing each group with the other groups. Interestingly, there appeared to be an overlap in the expression pattern of the isografts and the rejecting animals for IL-5, IL-1α, IL-18, and GRO-KC.

### Mediators relevant for classifications and progression of rejection

**Figure 4:**
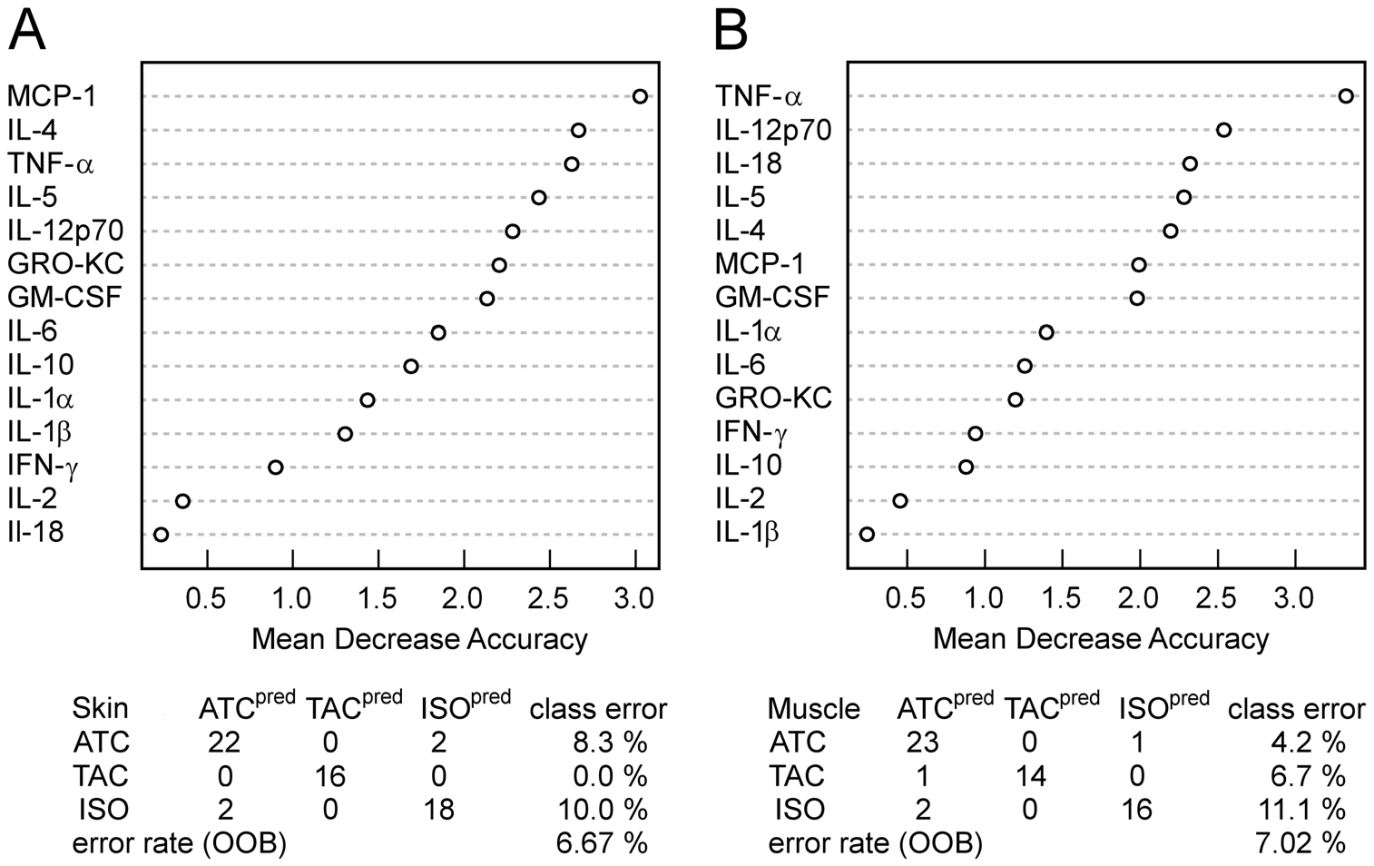
Results from Random Forest classification of the different rejection groups (ISO, TAC, ATC) using over the whole time course (POD 3, POD 5, POD 7, POD 9) measured inflammatory mediators in skin (A) and muscle (B) samples of rat limb transplantation models. Most important mediators for the decision trees based classification approach are evident by ranked mean decrease accuracy. Performances of the classifiers are indicated by the confusion table and the out-of-bag (OOB) error rate.

Random forest (RF) classification was performed for skin and muscle samples in all three cohorts at POD 3–9. The overall out-of-bag error rate was 6.7% for skin and 7.0% for muscle. The classification tables are shown in Figure 4. The importance of a variable in discriminating among study groups was demonstrated by ranked mean decrease accuracy as depicted in Figure 4. The mediator best capable of differentiating among experimental groups samples was MCP-1 in skin and TNF-α in muscle. Discriminant analysis suggested that the mediators best capable of discriminating among experimental groups in the early postoperative time points were GM-CSF, IL-4, IL-12p70, IL-5, and TNF-α; these mediators also appear within the seven top-ranked mediators in the RF analysis.

**Figure 5:**
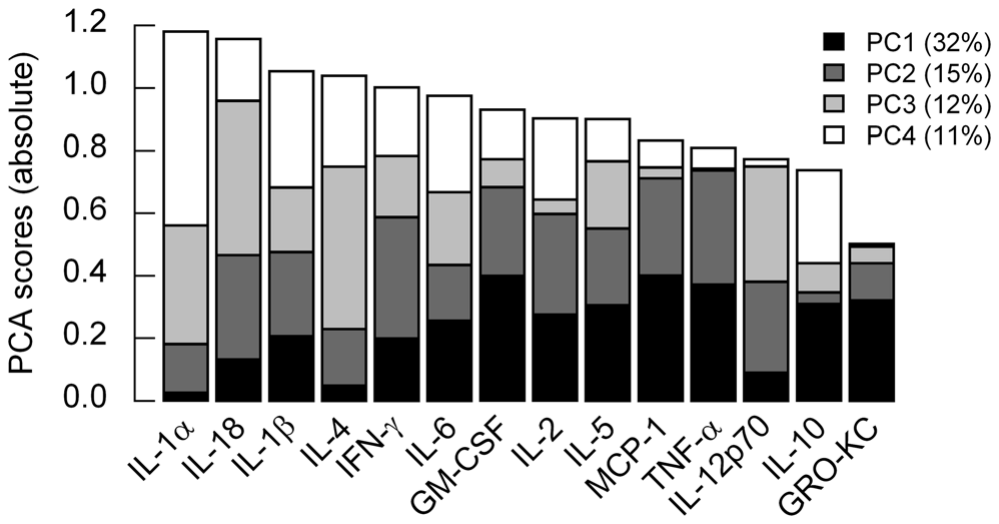
Most variable mediators identified by principal component analysis (PCA) suggesting new potential targets for therapeutic interventions to suppress limb transplant rejection. PCA scores (loadings) for the first four principal components (PCs), which represent more than 70% of information within the data, are displayed in a stacked bar plot for all inflammatory mediators (ranked by the overall PCA score of the 4 PCs).

To identify inflammatory cytokines with high dynamic variability, we performed principal component analysis (PCA) on the combined mean-centered dataset including the whole postoperative time series over all study groups and tissues. The PCA scores of the first four principle components (explaining >70% of the variance) of all 14 cytokines were ranked based on the sum of the absolute PCA scores (loadings) of all 4 PCs (Figure 5). The top prioritized cytokines IL-1α, IL-18, IL-1β, and IL-4 might be promising candidates for new therapeutic regimens.

## Discussion

Experimental transplant rejection can be detected reliably in advance of the current clinical gold standard of histologic evaluation, using computational modeling that involves 14 inflammatory mediators in this model. These findings support the hypothesis that the immune signaling associated with rejection follows specific patterns of expression and is driven by different principal components than those associated with inflammation following syngeneic transplantation.

Detailed cellular and molecular assessment provides valuable insights into the role of the immune/inflammatory response in post-transplant pathophysiology [14], [15]. Translation of these findings into biomarkers applied clinically, however, has been very limited. It remains a major challenge to reduce the complexity of dynamic biological systems to elements with diagnostic or therapeutic clinical value [16], [17]. Data-driven investigations of genomic and proteomic studies in combination with mechanistic computational modeling based on measurements of circulating inflammatory mediators have given insight into the pathophysiology of trauma, shock as well as organ transplantation [18]–[22].

Herein, we demonstrate that data-driven and expression pattern-oriented analyses of a high-content dataset can help to decipher the complexity of acute inflammation in VCA. In the present study, these advanced computational algorithms allowed to diagnose rejection in advance of gross histological damage. We suggest that measuring inflammatory mediators expressed in skin and muscle is clinically feasible in the setting of VCA since skin and muscle are more accessible as compared to solid organs. Isografts were compared to allografts and tacrolimus treated allografts in order to delineate between surgery/trauma/ischemia induced inflammation and allograft rejection. Tacrolimus treatment was sufficient to diminish rejection (but not completely eliminate the immune response).

In the samples analyzed in the present study, skin rejection was detected in advance of histological alterations based on the inflammatory mediators IL-4, IL-12p70 and TNF-α, with a prediction accuracy of >87%. In muscle rejection, IL-12p70 and TNF-α were identified as best and accurate classifiers. TNF-α, a canonical inflammatory mediator, is mainly produced by activated monocytes, macrophages, and T-cells, and exerts a direct effect on the proliferation, apoptosis, necrosis, differentiation, and function of virtually every cell type [23]. In the skin, mast cells appear to be the predominant source of preformed TNF-α, which can be released upon inflammatory stimuli [24]. TNF-α activates T-cells; increases the release of other inflammatory mediators; and induces neutrophil adherence, infiltration and the production of enzymes and reactive oxygen. These mechanisms are harmful to the allograft, and subsequently cause tissue injury as well as organ dysfunction [23]. Several studies have demonstrated increased TNF-α levels in the serum during episodes of rejection in liver, kidney and pancreas transplant recipients [25]–[27]. Interestingly, the increase in TNF-α levels occurred two days before clinical manifestation of rejection.

The production of IL-12 strongly promotes the development of IFN-γ producing helper T (TH)-cells. IL-12 may not only be essential for macrophage-mediated allograft rejection via induction of TH1 responses, but IL-12 may be involved in delaying rejection via induction of inducible nitric oxide synthase (iNOS) and indolamine 2,3 dioxygenase (IDO) [28]. This positive effect was shown in several skin or heart allograft models, as well as in the course of graft-versus-host disease (GVHD) [29]–[31].

Good performance of the multinomial logistic regression model, which models linear decision boundaries effectively, indicates that the patterns of inflammatory mediator expression in skin are fundamentally different in isograft and allograft skin tissue, and that this difference can be captured with reasonable performance by computationally efficient algorithms. Multinomial logistic regression performance for discrimination of rejection is even stronger and the consistency with which this distinction is made across a rather complex set of features implies a high level of biological significance. In other words, not only is the specific inflammatory mediator, but also the particular combination of mediators in the local inflammatory milieu seems to play a determinative role in the nature and progression of inflammation expressed.

The RF classification approach including more time points (POD 3-POD 9) achieves high levels of accuracy and continues to improve with the addition of variables. This finding implies that there are specific inflammatory mediator interactions that are relevant only under certain contexts [32], and that these interactions can be leveraged to identify potential targets for therapeutic intervention.

We hypothesized that mediators with time-dependent changes might be important in the different dynamic processes and describe principal drivers (principal components, PC), which could turn out as therapeutic targets. We were focusing on the first four PCs since they comprise more than 70% of the total variance. The four top-ranked mediators were IL-1α, IL-18, IL-1β, and IL-4 (Figure 5).

To discern the effect of the inflammatory mediators within each group, we performed the same PCA procedure for each group individually (Figure S1). The impact of IL12p70 already shown as potential early diagnostic marker for rejection was not as pronounced in the tacrolimus-treated animals as in the isograft and allograft study group. Interferon-γ and IL-4 had a high ranking in all groups (ISO, TAC, ATC) indicating a more unspecific effect in inflammation; In contrast, IL-1α and IL-18 exhibited an expression profile which indicates a possible key role in VCA rejection and makes these cytokines interesting candidates for therapeutic interventions.

Our studies identified IL-1α and IL-18 as possible candidates for the treatment of skin rejection. These inflammatory mediators share similarities regarding structure, receptor family, signal transduction pathways, and biological effects. Both inflammatory mediators are produced by monocytes/macrophages but also constitutively expressed by keratinocytes [33]. IL-1α plays an important role in sterile inflammation. During necrotic cell death, the IL-1α precursor is released [34] and binds to its receptor expressed on adjacent macrophages and epithelial cells, which in turn triggers a pro-inflammatory response characterized by an influx of neutrophils followed by macrophages [35], [36]. IL-18, together with IL-2, IL-12, and IL-15, is a dominant IFN-γ inducing factor. Several human diseases, such as systemic lupus erythematosus, rheumatoid arthritis, Crohn's disease, psoriasis and graft-versus-host-disease are thought to be mediated in part by IL-18 [37]. Moreover, IL-18 stimulates ICAM-1 expression on monocytic cell lines, which is important for the recruitment of T-cells and other immune cells to the skin. Lymphocyte recruitment is known as a key mechanism in inflammatory skin disorders and [38], [39]. Hautz et al. showed, that expression of ICAM-1 correlated closely with severity of skin rejection [8].

Based on our findings, IL-1α and IL-18 appear as interesting potential targets for intervention. Yuan J et al. already showed the efficacy of IL-1 receptor antagonist (IL-1ra) gene transfer treatment for acute corneal graft rejection in a rat model [40] The group demonstrated during acute rejection, that TGF-β1, RANTES and IL-1 levels were lower in the IL-1ra treatment group. Thus antagonizing the biological activitiy of IL-1 could effectively prolong graft survival. IL-ra, a specific inhibitor of both IL-1α and IL-β generically known as anakinra is clinically applied for the treatment of rheumatoid arthritis. IL-18-binding protein (IL-18BP), a specific inhibitor or IL-18 which neutralizes IL-18 bioactivity, was discovered during the search for soluble IL-18 receptors in humane urine [41]. A clinical preparation of human IL-18BP has been shown to be safe and effective in patients with RA or plaque psoriasis [42]. A soluble form of the IL-18 receptor accessory protein (sIL-18Rβ) has recently been identified as novel IL-18 inhibitor in collagen-induced arthritis in mice [43].

In summary, we herein provide information, which could help identifying a diagnostic profile and novel targets for treatment of skin rejection in VCA. The present study demonstrates that the application of advanced computational methods can be successfully applied in molecular assessment of skin rejection and provides novel insights into the inflammatory mediator communication patterns. The study remains observational in its nature and investigational trials are warranted in order to address the true functional value of the postulated treatment targets.

## Supporting Information

Figure S1 **Most variable (influential) mediators identified by principal component analysis (PCA) for each of the three study groups (ISO/TAC/ATC).** PCA scores (loadings) for the first four principal components (PCs), which represent more than 75% of information, are displayed in a stacked bar plot for all inflammatory mediators (ranked by the overall PCA score of the 4 PCs) and a scatter plot of the first two PCs.
(TIFF)

Figure S2 **Distribution of inflammatory mediator levels (boxplots) at postoperative day 5 in rat limb transplantation models for selected inflammatory mediators.** Adjusted p-values from Wilcoxon rank-sum test between the rejection group (ATC) versus Tacrolimus treated group (TAC) are provided.
(TIFF)

## References

[1] Cendales LC, Kanitakis J, Schneeberger S, Burns C, Ruiz P, et al (2008) The Banff 2007 working classification of skin-containing composite tissue allograft pathology. Am J Transplant 8: 1396–1400. 18444912 10.1111/j.1600-6143.2008.02243.x

[2] Kanitakis J, Jullien D, Nicolas JF, Frances C, Claudy A, et al (2000) Sequential histological and immunohistochemical study of the skin of the first human hand allograft. Transplantation 69: 1380–1385. 10798758 10.1097/00007890-200004150-00029

[3] Kanitakis J (2008) The challenge of dermatopathological diagnosis of composite tissue allograft rejection: a review. J Cutan Pathol 35: 738–744. 18422693 10.1111/j.1600-0560.2007.00889.x

[4] Schneeberger S, Gorantla VS, van Riet RP, Lanzetta M, Vereecken P, et al (2008) Atypical acute rejection after hand transplantation. Am J Transplant 8: 688–696. 18261182 10.1111/j.1600-6143.2007.02105.x

[5] Hautz T, Wolfram D, Grahammer J, Starzl R, Krapf C, et al.. (2012) Mechanisms and mediators of inflammation: potential models for skin rejection and targeted therapy in vascularized composite allotransplantation. Clin Dev Immunol: 1–9. 10.1155/2012/757310 PMC3459345 23049603

[6] Tarca AL, Carey VJ, Chen XW, Romero R, Draghici S (2007) Machine learning and its applications to biology. PLoS Comput Biol 3: e116. 17604446 10.1371/journal.pcbi.0030116 PMC1904382

[7] Coin L, Bateman A, Durbin R (2003) Enhanced protein domain discovery by using language modeling techniques from speech recognition. Proc Natl Acad Sci U S A 100: 4516–4520. 12668763 10.1073/pnas.0737502100 PMC404693

[8] Hautz T, Zelger B, Grahammer J, Krapf C, Amberger A, et al (2010) Molecular markers and targeted therapy of skin rejection in composite tissue allotransplantation. Am J Transplant 10: 1200–1209. 20353468 10.1111/j.1600-6143.2010.03075.x

[9] Benjamini Y, Hochberg Y (1995) Controlling the false discovery rate: A practical and powerful approach to multiple testing. J R Statist Soc B 57: 289–300.

[10] Sturn A, Quackenbush J, Trajanoski Z (2002) Genesis: cluster analysis of microarray data. Bioinformatics 18: 207–208. 11836235 10.1093/bioinformatics/18.1.207

[11] Breiman L (2001) Random Forests. Machine Learning 45: 5–32.

[12] Mi Q, Constantine G, Ziraldo C, Solovyev A, Torres A, et al (2011) A dynamic view of trauma/hemorrhage-induced inflammation in mice: principal drivers and networks. PLoS One 6: e19424. 21573002 10.1371/journal.pone.0019424 PMC3091861

[13] Namas RA, Namas R, Lagoa C, Barclay D, Mi Q, et al (2012) Hemoadsorption reprograms inflammation in experimental gram-negative septic peritonitis: insights from in vivo and in silico studies. Mol Med 18: 1366–1374. 22751621 10.2119/molmed.2012.00106 PMC3533640

[14] Page EK, Dar WA, Knechtle SJ (2012) Biologics in organ transplantation. Transpl Int 25: 707–719. 22420711 10.1111/j.1432-2277.2012.01456.x

[15] Sarwal MM (2006) Chipping into the human genome: novel insights for transplantation. Immunol Rev 210: 138–155. 16623769 10.1111/j.0105-2896.2006.00359.x

[16] Vodovotz Y (2006) Deciphering the complexity of acute inflammation using mathematical models. Immunol Res 36: 237–245. 17337784 10.1385/IR:36:1:237

[17] Mesarovic MD, Sreenath SN, Keene JD (2004) Search for organising principles: understanding in systems biology. Syst Biol (Stevenage) 1: 19–27. 17052112 10.1049/sb:20045010

[18] Calvano SE, Xiao W, Richards DR, Felciano RM, Baker HV, et al (2005) A network-based analysis of systemic inflammation in humans. Nature 437: 1032–1037. 16136080 10.1038/nature03985

[19] Warren HS, Elson CM, Hayden DL, Schoenfeld DA, Cobb JP, et al (2009) A genomic score prognostic of outcome in trauma patients. Mol Med 15: 220–227. 19593405 10.2119/molmed.2009.00027 PMC2707513

[20] Chow CC, Clermont G, Kumar R, Lagoa C, Tawadrous Z, et al (2005) The acute inflammatory response in diverse shock states. Shock 24: 74–84. 15988324 10.1097/01.shk.0000168526.97716.f3

[21] Bohra R, Klepacki J, Klawitter J, Thurman JM, Christians U (2013) Proteomics and metabolomics in renal transplantation-quo vadis? Transpl Int 26: 225–241. 23350848 10.1111/tri.12003 PMC4006577

[22] Azhar N (2013) Analysis of serum inflammatory mediators identifies unique dynamic networks associated with death and spontaneous survival in pediatric acute liver failure. 10.1371/journal.pone.0078202 PMC3823926 24244295

[23] Grenz A, Schenk M, Zipfel A, Viebahn R (2000) TNF-alpha and its receptors mediate graft rejection and loss after liver transplantation. Clin Chem Lab Med 38: 1183–1185. 11156356 10.1515/CCLM.2000.184

[24] Walsh LJ, Trinchieri G, Waldorf HA, Whitaker D, Murphy GF (1991) Human dermal mast cells contain and release tumor necrosis factor alpha, which induces endothelial leukocyte adhesion molecule 1. Proc Natl Acad Sci U S A 88: 4220–4224. 1709737 10.1073/pnas.88.10.4220 PMC51630

[25] Imagawa DK, Millis JM, Olthoff KM, Derus LJ, Chia D, et al (1990) The role of tumor necrosis factor in allograft rejection. I. Evidence that elevated levels of tumor necrosis factor-alpha predict rejection following orthotopic liver transplantation. Transplantation 50: 219–225. 2382288 10.1097/00007890-199008000-00009

[26] Bubnova LN, Kabakov A, Serebrianaya N, Ketlinky S (1992) Interleukin-1 beta and tumor necrosis factor-alpha serum levels in renal allograft recipients. Transplant Proc 24: 2545. 1465860

[27] Grewal HP, Kotb M, Salem A, el Din AB, Novak K, et al (1993) Elevated tumor necrosis factor levels are predictive for pancreas allograft transplant rejection. Transplant Proc 25: 132–135. 7679807

[28] Goriely S, Goldman M (2008) Interleukin-12 family members and the balance between rejection and tolerance. Curr Opin Organ Transplant 13: 4–9. 18660699 10.1097/MOT.0b013e3282f406c4

[29] Piccotti JR, Chan SY, Goodman RE, Magram J, Eichwald EJ, et al (1996) IL-12 antagonism induces T helper 2 responses, yet exacerbates cardiac allograft rejection. Evidence against a dominant protective role for T helper 2 cytokines in alloimmunity. J Immunol 157: 1951–1957. 8757314

[30] Verma ND, Boyd R, Robinson C, Plain KM, Tran GT, et al (2006) Interleukin-12p70 prolongs allograft survival by induction of interferon gamma and nitric oxide production. Transplantation 82: 1324–1333. 17130782 10.1097/01.tp.0000239519.56358.c1

[31] Dey BR, Yang YG, Szot GL, Pearson DA, Sykes M (1998) Interleukin-12 inhibits graft-versus-host disease through an Fas-mediated mechanism associated with alterations in donor T-cell activation and expansion. Blood 91: 3315–3322. 9558388

[32] Nathan C, Sporn M (1991) Cytokines in context. J Cell Biol 113: 981–986. 2040651 10.1083/jcb.113.5.981 PMC2289009

[33] Dinarello CA (1999) IL-18: A TH1-inducing, proinflammatory cytokine and new member of the IL-1 family. J Allergy Clin Immunol 103: 11–24. 9893178 10.1016/s0091-6749(99)70518-x

[34] Carmi Y, Voronov E, Dotan S, Lahat N, Rahat MA, et al (2009) The role of macrophage-derived IL-1 in induction and maintenance of angiogenesis. J Immunol 183: 4705–4714. 19752225 10.4049/jimmunol.0901511

[35] Rider P, Carmi Y, Guttman O, Braiman A, Cohen I, et al (2011) IL-1alpha and IL-1beta recruit different myeloid cells and promote different stages of sterile inflammation. J Immunol 187: 4835–4843. 21930960 10.4049/jimmunol.1102048

[36] van de Veerdonk FL, Netea MG (2013) New Insights in the Immunobiology of IL-1 Family Members. Front Immunol 4: 167. 23847614 10.3389/fimmu.2013.00167 PMC3703542

[37] Dinarello CA (2009) Immunological and inflammatory functions of the interleukin-1 family. Annu Rev Immunol 27: 519–550. 19302047 10.1146/annurev.immunol.021908.132612

[38] Robert C, Kupper TS (1999) Inflammatory skin diseases, T cells, and immune surveillance. N Engl J Med 341: 1817–1828. 10588968 10.1056/NEJM199912093412407

[39] Schon MP, Ludwig RJ (2005) Lymphocyte trafficking to inflamed skin—molecular mechanisms and implications for therapeutic target molecules. Expert Opin Ther Targets 9: 225–243. 15934912 10.1517/14728222.9.2.225

[40] Yuan J, Liu Y, Huang W, Zhou S, Ling S, et al (2013) The experimental treatment of corneal graft rejection with the interleukin-1 receptor antagonist (IL-1ra) gene. PLoS One 8: e60714. 23723965 10.1371/journal.pone.0060714 PMC3665808

[41] Novick D, Kim SH, Fantuzzi G, Reznikov LL, Dinarello CA, et al (1999) Interleukin-18 binding protein: a novel modulator of the Th1 cytokine response. Immunity 10: 127–136. 10023777 10.1016/s1074-7613(00)80013-8

[42] Tak PP, Bacchi M, Bertolino M (2006) Pharmacokinetics of IL-18 binding protein in healthy volunteers and subjects with rheumatoid arthritis or plaque psoriasis. Eur J Drug Metab Pharmacokinet 31: 109–116. 16898079 10.1007/BF03191127

[43] Veenbergen S, Smeets R, Bennink M, Arntz O, Joosten L, et al (2010) The natural soluble form of IL-18 receptor beta exacerbates collagen-induced arthritis via modulation of T-cell immune responses. Ann Rheum Dis 69: 276–283. 19188194 10.1136/ard.2008.100867

