# Supplementary figures and images for: Insights from Computational Modeling in Inflammation and Acute Rejection in Limb Transplantation
Source: PLoS One. 2014 Jun 13;9(6):e99926. doi: 10.1371/journal.pone.0099926 (PMC4057425; doi:10.1371/journal.pone.0099926)

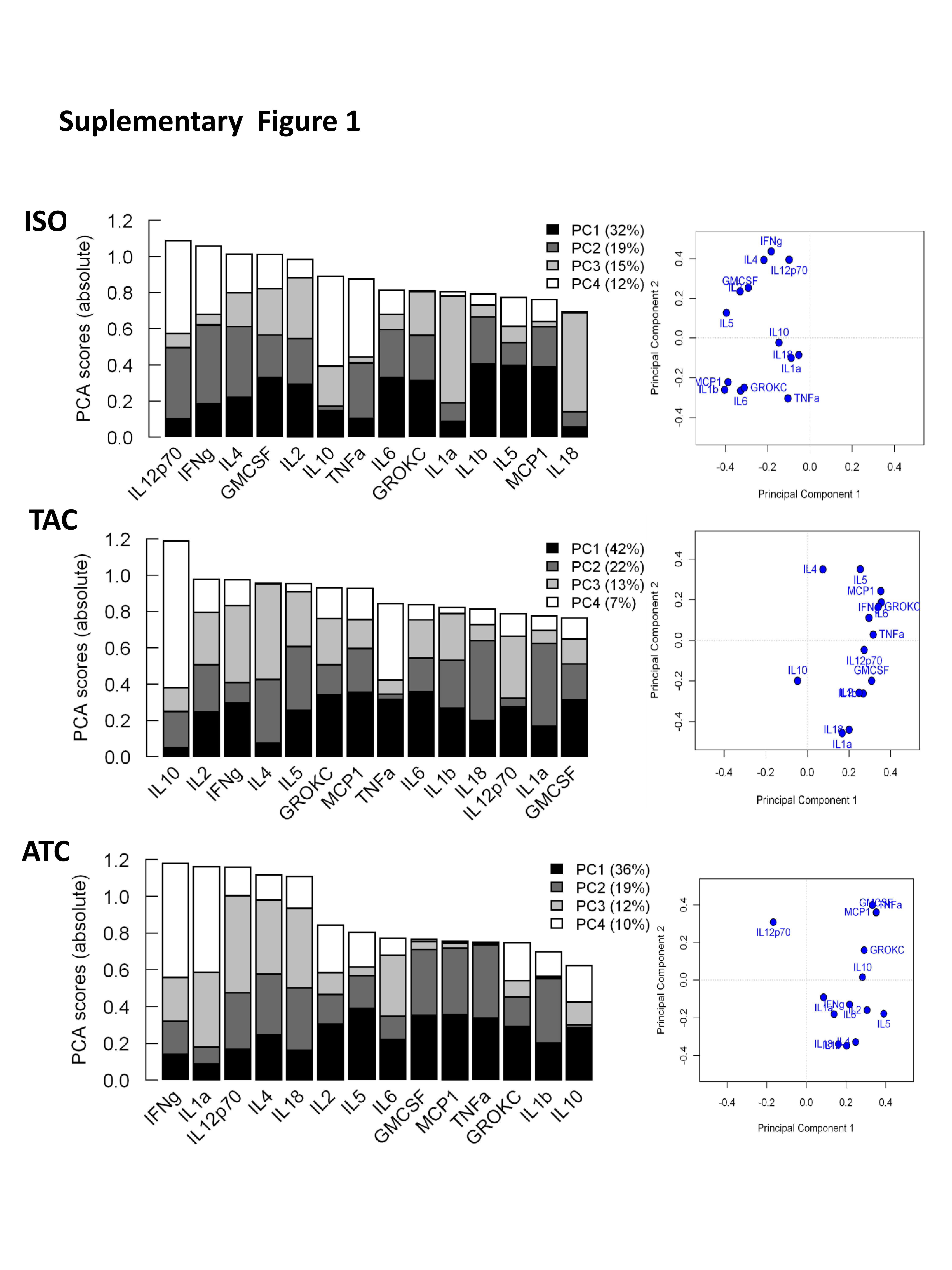

Supplement: Figure S1 — Most variable (influential) mediators identified by principal component analysis (PCA) for each of the three study groups (ISO/TAC/ATC). PCA scores (loadings) for the first four principal components (PCs), which represent more than 75% of information, are displayed in a stacked bar plot for all inflammatory mediators (ranked by the overall PCA score of the 4 PCs) and a scatter plot of the first two PCs. (TIFF) [file pone.0099926.s001.tiff]

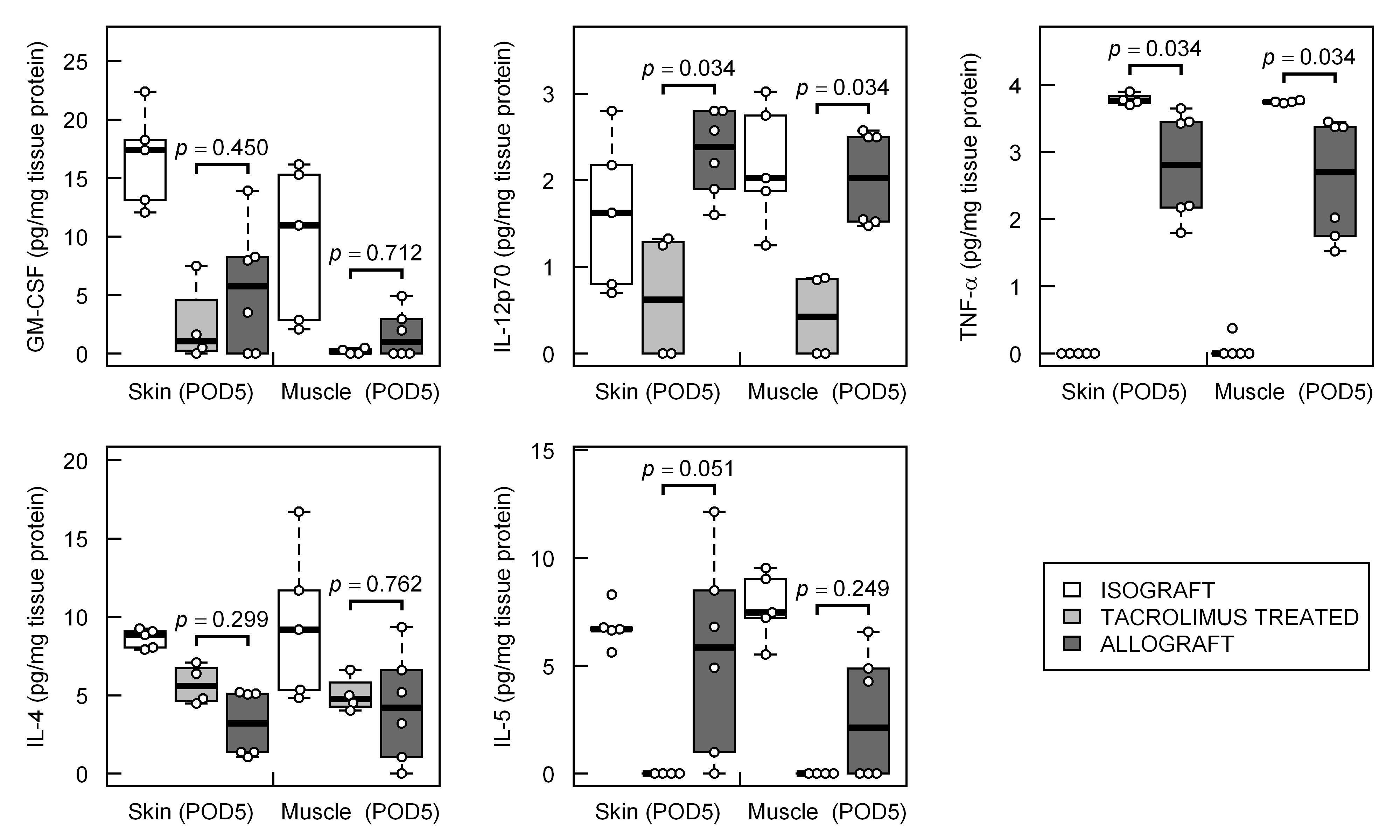

Supplement: Figure S2 — Distribution of inflammatory mediator levels (boxplots) at postoperative day 5 in rat limb transplantation models for selected inflammatory mediators. Adjusted p-values from Wilcoxon rank-sum test between the rejection group (ATC) versus Tacrolimus treated group (TAC) are provided. (TIFF) [file pone.0099926.s002.tiff]
